# Low-Cost Conversion of Single-Zone HVAC Systems to Multi-Zone Control Systems Using Low-Power Wireless Sensor Networks

**DOI:** 10.3390/s20133611

**Published:** 2020-06-27

**Authors:** Julio Antonio Jornet-Monteverde, Juan José Galiana-Merino

**Affiliations:** 1 Department of Physics, Systems Engineering and Signal Theory, University of Alicante, Crta. San Vicente del Raspeig, s/n, 03080 Alicante, Spain; julio.jornet@ua.es; 2 University Institute of Physics Applied to Sciences and Technologies, University of Alicante, Crta. San Vicente del Raspeig, s/n, 03080 Alicante, Spain

**Keywords:** wireless sensor networks, Internet of Things (IoT) platforms, Wi-Fi networks, CC3200, heating, ventilation and air conditioning (HVAC) systems, Message Queuing Telemetry Transport (MQTT)

## Abstract

This paper presents a novel approach to convert a conventional house air conditioning installation into a more efficient system that individually controls the temperature of each zone of the house through Wi-Fi technology. Each zone regulates the air flow depending on the detected temperature, providing energy savings and increasing the machine performance. Therefore, the first step was to examine the communication bus of the air conditioner and obtain the different signal codes. Thus, an alternative Controller module has been designed and developed to control and manage the requests on the communication bus (Bus–Wi-Fi gateway). A specific circuit has been designed to adapt the signal of the serial port of the Controller with the communication bus. For the acquisition of the temperature and humidity data in each zone, a Node module has been developed, which communicates with the Controller through the Wi-Fi interface using the Message Queuing Telemetry Transport (MQTT) protocol with Secure Sockets Layer / Transport Layer Security (SSL/TLS) certificates. It has been equipped with an LCD touch screen as a human-machine interface. The Controller and the Node modules have been developed with the ultra-low power consumption CC3200 microController of Texas Instruments and the code has been implemented under the TI-RTOS real-time operating system. An additional module based on the Raspberry Pi computer has been designed to create the Wi-Fi network and implement the required network functionalities. The developed system not only ensures that the temperature in each zone is the desired one, but also controls the fan velocity of the indoor unit and the opening area of the vent registers, which considerably improves the efficiency of the system. Compared with the single-zone system, the experiments carried out show energy savings between 75% and 94% when only one of the zones is selected, and 44% when the whole house is air-conditioned, in addition to considerably improving user comfort.

## 1. Introduction

Due to climate change and population growth, the demand for energy in buildings around the world is growing considerably. Concretely, 30% of global final energy use and 28% carbon dioxide (CO_2_) emissions in 2017 were accounted by buildings [1]. In addition, most of this energy (about 40%) was consumed in heating, ventilation and air conditioning (HVAC) systems [1]. 

In the case of space heating, the energy sources used are more diverse: electricity, gas, solid fuels, petroleum products, and, increasingly, renewable energy. Besides, advances in the thermal insulation of buildings are helping to effectively reduce the energy consumption. In fact, from 2010 to 2017, the energy consumption in space heating was reduced by approximately 18% [1]. 

In contrast, the electricity consumed in space cooling has been growing considerably in recent years, especially in the world’s hotter regions. The energy dedicated to space cooling has increased by 6% from 2010 to 2017 [1], and currently represents approximately 20% of the total electricity used in buildings around the world and 10% of all global electricity consumption [2]. At this rate, these percentages may be transformed into 40% and 20% in 2050, respectively [2].

It is clear that urgent actions are needed to improve the efficiency of HVAC systems. These actions may include the use of passive cooling techniques, more energy efficient equipment, multi-zone management systems or advanced humidity and temperature control systems.

Passive cooling techniques are the most energy efficient as they use electric fans and natural air currents to cool indoor air (e.g., [3,4,5,6,7,8]). However, the effectiveness of these techniques depends on the local weather conditions at each site. For example, maximum daytime temperatures of up to 45 °C and nighttime temperatures of 25 °C were reached in some regions of Southern Europe during the summer of 2019 [9], making passive cooling ventilation completely inefficient to maintain an adequate indoor temperature. In these situations, air conditioner systems are mandatory. 

More efficient air conditioner equipment may reduce the cooling energy demand by 45% regarding the expected scenario for 2050 [2]. Currently, however, the average efficiency of air conditioners sold is three times below the best available technology [2]. Therefore, in addition to technological advancement, new effective policies should be required. 

Multi-zone management systems are another alternative way to save electricity (e.g., [10,11]). In this line, most works in energy management systems are applied in non-residential buildings (e.g., university campus [12], office building [13], kindergarten school [14], etc.), providing device connectivity and independent automatic temperature control for each of the different zones. 

However, single-zone HVAC systems are installed in most residential buildings, which implies regulating all the rooms of the house with a single thermostat and with static ventilation registers. In these systems, air flow is inefficiently distributed through all rooms without maintaining a comfortable temperature in each of them and without taking into account their occupancy. The high price of commercial multi-zone systems ensures that almost all residential buildings have single-zone systems.

Finally, energy efficiency and energy rationalization algorithms are also incorporated in some zoning systems, providing a more responsible use of energy. In this sense, different strategies can be found in the literature for passive and mechanical cooling systems. These techniques include simple on/off control systems (e.g., [15]) to more advanced control techniques based on artificial neural networks (e.g., [16]), fuzzy logic (e.g., [17]), genetic algorithms (e.g., [18]), etc. A complete overview of the control methods used in HVAC systems can be found in the work of Afram and Janabi-Sharifi [19].

In this paper, the energy efficiency in residential houses is tackled by developing a low-cost prototype that enables converting a single-zone HVAC system into a multi-zone one.

From 2014 to 2018, only in Spain, about three million air conditioning units were installed in the residential sector, increasing approximately by 12% compared to 2017 [20]. Taking into account the number of houses in 2018 (about 18.5 million houses [21]) and the number of units installed in the last ten years, it can be estimated that air conditioning equipment is installed in approximately 35% of Spanish homes. In most of them, the complete house is covered by a single unit with only one thermostat, which provides the same air flow for all the rooms. This influences the comfort of the occupants negatively, in addition to being completely energy inefficient. By improving the installed HVAC systems, the electricity demand might be significantly reduced, especially in the summer season. For example, in the capital of Spain, Madrid, a third of the total electricity consumption can be accounted to air conditioners at times of greatest demand in summer [22].

Multi-zone systems are designed to provide individual monitoring of the temperature in each of the zones and control accordingly the air conditioning machine and the respective vent registers. These functions fit perfectly inside the ever-growing Internet of Things (IoT), which provides the tools required for developing wireless sensor networks (WSNs).

Energy management systems for non-residential buildings are dealt with in most of the works found in the literature (e.g., university campus [12], office building [13], kindergarten school [14], etc.). In these cases, an independent automatic temperature control for each of the different zones is carried out using techniques based on artificial neural networks (e.g., [16]), fuzzy logic (e.g., [17]), genetic algorithms (e.g., [18]), etc.

The problem addressed in these works is a bit different than the one addressed in this paper. It is well known that the individual energy consumption of any house is much lower than the one obtained in a building, university campus, shopping center, etc. However, when considering residential buildings as a whole, the amount of energy consumed increases considerably and should be taken into consideration.

In this regard, some papers can be found in the literature related to the retrofitting of the HVAC systems in residential buildings using IoT technologies (e.g., [10,23,24,25]). In the work of Redfern et al. [23], a vent register control system for residential houses was proposed. In this case, the designed WSN was controlled through a Java server (operating on a Linksys NSLU2) and the user interface is through an Internet Personal Home Page (PHP) webpage. However, the simple air flow restriction through the vent registers, without any operation on the air conditioning unit, might create backpressure in the ducts, producing leaks in the ducts and at the dampers. Besides, no experimentation was shown regarding operation or possible energy savings.

The bases of the work of Watts et al. [24] are similar to those of Redfern et al. [23], based on the vent register control. In this case, a multi-zone HVAC control system was applied for heating a two-story house over three days. The energy consumption for the whole house was compared with the one for each of the selected zones, obtaining energy savings between 50% and 94%.

A ZigBee protocol-based multi-zone HVAC control system was developed by Li et al. [25] for a residential house. In this case, connecting directly the control terminals of the wireless HVAC device to the heater-fan equipment, the temperature was controlled. The system was tested by simulation assuming a two-story house with 12 rooms and it was estimated a 27.8% energy saving for heating and 38.6% for cooling.

In the work of Sookoor et al. [10], the viability of retrofitting a centralized HVAC system was also analyzed. It was concluded that room zoning is effective even if the air conditioning system was initially designed for the entire house, what is the situation dealt in the present paper. A WSN composed by 21 temperature sensors and seven wirelessly controlled vent registers was proposed. The HVAC equipment was turned off or on depending on the set-point temperature of an Internet-controllable thermostat, but there was no direct control over the equipment. Although this operation mode was not the most efficient, they obtained a 20.5% reduction in the energy consumption over a 20-day experimental period. 

Finally, an automatic control system was proposed by Rajith et al. [13] to act on several individually controlled indoor air conditioning units. Although this is not the situation analyzed directly in this article, it is the situation of many other homes, which lack centralized air conditioning and therefore have installed one or more split air conditioner units. A predictive model based on artificial neural networks was used for optimizing the HVAC control. Their experiments were performed in an office space during summer season, achieving 20%–40% saving in energy consumption approximately.

To summarize, most of the systems applied on residential buildings present the following common characteristics:

- Their performance is based on the control of the vent registers, just opening or closing them. However, as was commented previously, the simple air flow restriction through the vent registers, without any operation on the air conditioning unit, might create overpressure in the ducts. In the work of Li et al. [25], the heater-fan equipment is controlled by the developed system, just connecting the required analog terminals.
- The performance of the commented systems, in terms of energy savings, sometimes is not presented or is based on simulations. Only some of them analyze real examples.
- In many of these works, an exhaustive description of the proposed development is lacking, for both hardware and software, which prevents its reproducibility. In the work of Mataloto et al. [14], the characteristics of different building and energy management systems are summarized, and it can be observed that almost all of them are commercial or not available. Besides, the control or operation of the indoor machine is always assumed without specifying anything about the bus or the protocol used by the air conditioning system.
- In the case of non-residential buildings, sophisticated controls can be found in the literature e.g., [16,17,18]. However, they are out of the requirements for the most of the common residential houses. It is true that these automatic Controllers can help to improve the energy efficiency of the HVAC system, but users want a simple, user friendly interface that allow them to switch on/off the air flow and control the desired temperature in each of the rooms, according to their comfortable levels, which are different for each person and cannot be measured only in terms of occupancy, light, temperature, humidity, etc.

In the present work, a low-cost and user-friendly system has been developed to transform an existing single-zone HVAC installation with constant air volume (CAV) for the house into a multi-zone HVAC system with variable air volume (VAV). There are four main contributions of the article. First, the indoor machine is directly controlled by the proposed system through its communication bus, which allows modifying the fan velocity and the set-point temperature of the indoor unit. The design and implementation of the complete proposed system, from the connection with the existing air conditioning bus to the monitoring of the temperature sensors, are explained in detail. Second, both the indoor unit and the vent registers (with four different opening degrees) are managed by the developed temperature control algorithm. The developed software and the temperature control algorithm are also explained. Third, a custom communication structure has been designed for the WSN based on the Message Queuing Telemetry Transport (MQTT) protocol. The details of the communication and the structure of the established messages are discussed. Finally, thanks to the use of commercial off-the-shelf components and open-source software, any researcher might reproduce the proposed system and adapt it to their own requirements (e.g., different number of zones, different temperature control algorithms, etc.). Besides, the proposed multi-zone conversion is accessible to any user since it is a low-cost and easy to implement system which does not require additional masonry or electricity works. 

Regarding the system evaluation, simulations and real experiments have been carried out to analyze the performance of the complete system and evaluate the energy consumption compared with the single-zone mode.

The designed WSN is based on the Raspberry Pi computer (RPI Broker) [26] and the Texas Instruments (TI) CC3200 platform [27], which constitutes a low-cost and low-power solution for IoT applications, with Wi-Fi connectivity and multiple Internet protocols (TCP/IP, SSL/TLS, HTTP, etc.). In our case, the Wi-Fi technology and the MQTT protocol [28], with AES256 encryption, have been used. This protocol uses a publisher subscriber philosophy, characterized by a lightweight implementation and a limited operating bandwidth, what makes it suitable for this context [28,29]. The developed prototype has been implemented and tested in a real environment using the indoor FDUR-258A unit of Mitsubishi Electric Corporation.

The rest of this paper is organized as follows. Section 2 describes in detail the hardware and software implementation of the developed prototype, as well as the temperature control algorithm. Section 3 presents the physical implementation and the technical characteristics of the system, as well as different performance tests. Finally, in Section 4, the main conclusions and future works are commented upon. 

## 2. Materials and Methods

### 2.1. Model Description

The majority of air conditioning systems are based on two units: the first is an outdoor unit, which contains the compressor, the condenser and the expansion valve; and the second unit, which is located inside (indoor unit), handles the exchange of temperature and ventilation in the system by air conduction. Normally the indoor unit is placed on the ceiling of the bathroom.

These types of system have the main board in the indoor air conditioning unit and the control panel placed in the living room or main room of the house. The control panel, operating in master mode, is connected to the main board through a three-wire communication bus.

These devices are usually prepared to support a second control panel, which would connect to the same communication bus in slave mode. Considering this possibility, a wireless control system linked to the secondary control panel has been designed in order to convert the commonly installed single-zone HVAC system into a multi-zone one. The developed system allows configuring, starting and stopping the air conditioning unit from any room in the house. For that, a gateway has been designed to receive different orders from different rooms and adapt them to the manufacturer’s communications bus.

**Figure 1:**
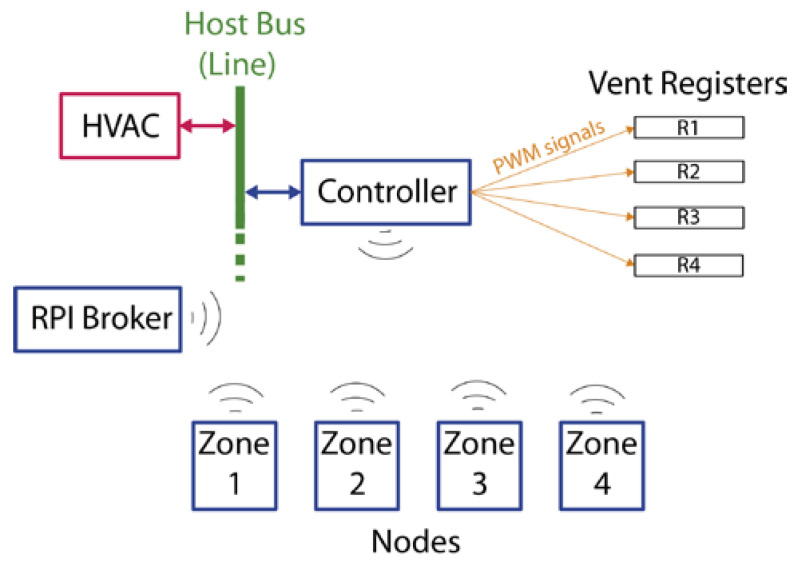
General scheme of the developed system and its elements.

The designed system consists of a unit called Controller, which works as a gateway and is physically connected to the communications bus; and four remote units called Nodes, which control the temperature and humidity in each of the rooms. The communication between the Controller and the Nodes is via Wi-Fi. Moreover, there is an additional module based on the Raspberry Pi computer [26] (RPI Broker), which implements the functionality of the MQTT Broker [28], the Network Time Protocol (NTP) server [30,31], and the Open Home Automation Bus (OpenHab) server [32], and also works as access point for the created Wi-Fi network. Figure 1 shows the general scheme of the developed system and its components.

The air flow for each one of the zones is controlled by the corresponding Node through a motorized vent register, which regulates its aperture depending on the temperature in the room. To facilitate the installation of the proposed system and avoid chasing in walls, the wiring to the vent registers is made directly through the inside of the air ducts. 

**Figure 2:**
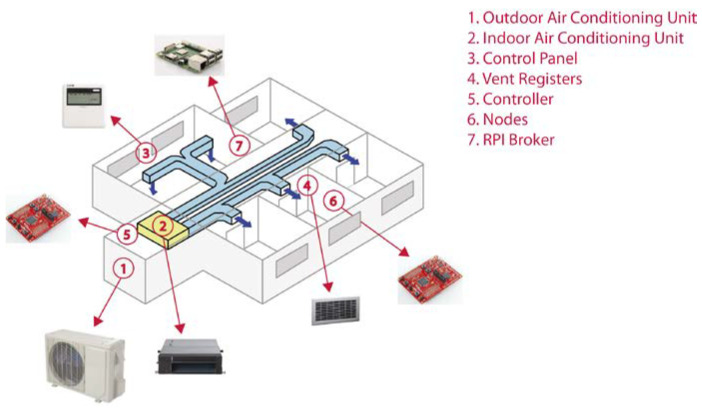
Example of distribution of the main elements of the proposed system.

Figure 2 shows the distribution of the main elements of the HVAC system, taking as a reference a house with one dining/living room, one kitchen, one bathroom and three bedrooms. In the proposed system, the Controller is located close to the indoor air conditioning unit and connected to the communication bus. In each of the rooms, a Node unit is installed to measure the temperature and the humidity and control the motorized vent registers accordingly. Finally, the RPI Broker can be placed in any room, although preferably in a centered place of the house to provide the maximum Wi-Fi coverage to all the Nodes.

The Texas Instruments (TI) CC3200 platform [27] has been used for the development of the Controller and the Nodes due to its low power consumption and the integration of the Wi-Fi interface. Specifically, the CC3200 LaunchPad development board has been chosen.

The software has been developed using the compiler Code Composer Studio (CSS) [33] together with the Software Development Kit (SDK) [34] recommended by Texas Instruments for the CC3200 programming. The Texas Instrument Real-Time Operating System (TI-RTOS) has been used, since includes a real-time multitasking management together with additional middleware components (e.g., device drivers). In our case, the developed program has been designed to make use of the multitasking characteristics, so several tasks will work in parallel.

### 2.2. Controller

The complete operation of the Controller is basically performed by two tasks, whose main functions are:

1. Main task:
  - Serial bus synchronization (host bus);
  - Sending/receiving messages to the serial bus;
  - Controlling the timer;
  - Opening/closing of the vent registers;
  - Command Line Interface (CLI).
2. WLAN (Wireless Local Area Network) task:
  - Controlling the Wi-Fi and NTP connection.

For the controlling MQTT protocol, three interrupts have been also configured with the following characteristics:

1. Power Reset and Clock Module (PRCM). It is the main timer of the program and occurs every second.
2. Command Line Interface (CLI). It is activated every time that the UART0 receives a character. This interrupt is used as an aid to the development and troubleshooting of the code. A library has been developed with a series of commands that allow displaying the status of each Node/Controller and the value of the variables used. Besides, the Wi-Fi selection, SSID (Service Set Identifier) and password parameters, can be also configured through one of these commands, either for the first time or because a change network is required.
3. Serial Bus: This is activated every time that the UART1 (Universal Asynchronous Receiver-Transmitter) receives a character. It is used to connect the UART1 with the host bus.

The Controller is connected to one temperature sensor, several servo motors, and one line voltage adapter circuit.

**Figure 3:**
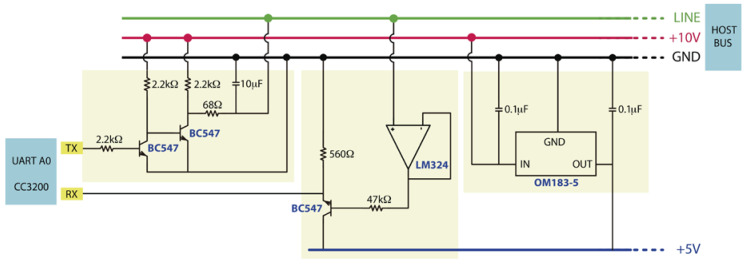
Line voltage adapter circuit.

- One of the GPIO pins of the CC3200 LaunchPad is connected to the Data Pin of a digital temperature and humidity sensor. In our case, the DHT22 [35] sensor has been used, which operates between –40 and 80 °C with a resolution of 0.1 °C and accuracy lower than ±0.5 °C.
- Each of the PWM (Pulse-Width Modulation) outputs of the Controller are connected to a servo motor, (e.g., MG996R Digital Servo [36]) to control the opening and closing of the corresponding vent registers. The CC3200 microController offers 4 PWM outputs. However, the number of PWM outputs might be extended to 16 by using a PCA9685 model [37].
- The RX and TX pins (UART1) are connected to a line voltage adapter circuit, which have been designed to adapt the UART1 signals (from 0 to 3.3 V) to the line signals of the host bus (from 0 to 10 V, respectively). Figure 3 shows the designed circuit.

In both devices, the Controller and the Nodes, the CC3200 LaunchPad includes three LEDs that are used as status indicators:

- A green LED, which blinks every second indicating the right functioning;
- A yellow LED that indicates the right Wi-Fi connection;
- A red LED, which blinks every time that the microController detects a message in the host bus.

#### 2.2.1. Serial Bus and Communication Characteristics

The communication is established through messages structured with different fields or variables. In general, some of the most important variables involved in the communication process are:

1. Device identification. Master, slave, main board.
2. Power status and working mode: AUTO, DRY, COOL, FAN, or HEAT.
3. Output_Temp. This is the selected temperature at the air outlet of the indoor unit or at the control panel.
4. Fan level. This parameter indicates the desired ventilation velocity in the indoor air conditioning unit (e.g., low, middle and high).
5. Return_Temp. This is the temperature measured by the control panel or the main board in the returned air of the ceiling.

**Figure 4:**
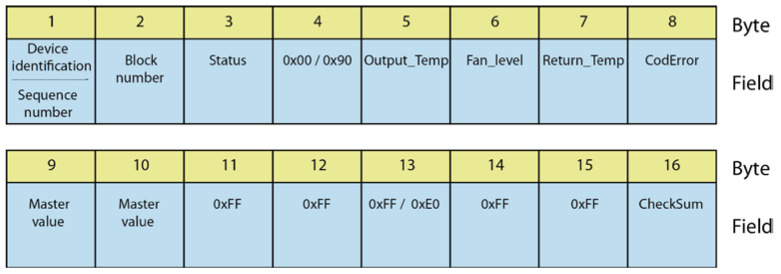
Example of message structure.

In Figure 4, an example of a 16-byte message is shown.

For the communication with the host bus, the UART1 interrupt is used. According to the specifications of CC3200, this interrupt can be configured to trigger every time a character or a set of characters (maximum seven) is received. For that, the UART1 is provided with an 8-character buffer. 

In our case, the most efficient configuration is the one that activates the interrupt after seven characters. In other way, the program execution would become much slower, interrupting all tasks every time a character arrives.

At the beginning of the communication, the interrupt is activated with any received character in order to examine the correct reception of the messages. From the moment 16 characters in a row with the correct checksum value are received, then the 16-byte messages will be clearly identified, and the interrupt configuration will be changed to trigger every seven characters. Although the interrupt is activated with the seventh character, the buffer reading is delayed for 10 ms to fully read the eight characters of the buffer. Thus, the interrupt will be activated twice for each message. 

If the message synchronization were lost during any moment of the communication (e.g., due to a line error), the program would change the UART1 interrupt to the character-to-character configuration and repeat the previous commented synchronization process. The loss of a frame is controlled by the PRCM interrupt, which is the main timer of the program and is triggered every second.

In the character-to-character mode, the TimerA2 interrupt is enabled and configured to trigger every 30 ms. Each time a character is received, the TimerA2 is restarted. However, if no character is received during 30 ms, then the interrupt will be activated, and the synchronization process will start again. At the moment the program returns to the seven-character mode, the TimerA2 interrupt will be disabled.

### 2.3. Nodes

The complete operation of the Nodes is basically performed by three tasks, whose main functions are:

1. Main task:
  - Controlling the main timer;
  - Controlling the room temperature;
  - Command Line Interface (CLI);
  - Controlling the Low-Power Deep Sleep (LPDS) mode.
2. WLAN task:
  - Controlling the Wi-Fi and NTP connection;
  - Controlling MQTT protocol.
3. LCD task:
  - Controlling the LCD/touch interface.

Four interrupts have been also configured with the following characteristics:

1. TIMER_A0. This is the main timer of the program in the active mode and occurs every second.
2. PRCM. This is the main timer of the program in the LPDS mode and occurs every 30 s.
3. CLI. This is activated every time that the UART0 receives a character. This is the same commented previously for the Controller.
4. Touch. This is activated every time that the capacitive LCD touch screen is touched.

Each one of the Nodes is connected to one temperature sensor and one LCD touch screen. In our case, the digital temperature and humidity sensor used, i.e., DHT22 [35], is connected to one of the GPIO pins of the CC3200 LaunchPad. Regarding the characteristics and connections of the LCD touch screen, these are explained in the next section. 

#### 2.3.1. LCD Touch Screen Interface

The Nodes are connected to a 2.8 ’’ TFT LCD (Thin Film Transistor-Liquid Crystal Display) screen (240 × 320 dots) equipped with a capacitive touch panel. The selected model has been the ER-TFTM028-4 [38] that uses the ILI9341 chip as the TFT LCD Controller and the FT6206 chip as the capacitive touch Controller.

The SPI (Serial Peripheral Interface) bus has been used to refresh the TFT LCD screen using the maximum possible clock frequency, i.e., 20 MHz. The I^2^C (Inter-Integrated Circuit) bus has been used to retrieve the capacity values of the touch screen at the frequency clock of 2 MHz.

The libraries provided by the peripheral manufacturer have been used for the graphic interface. Data matrices have been defined for creating the different fonts and icons. To reduce the memory size, both the symbols and the fonts are drawn in a single color (white on blue background). 

**Figure 5:**
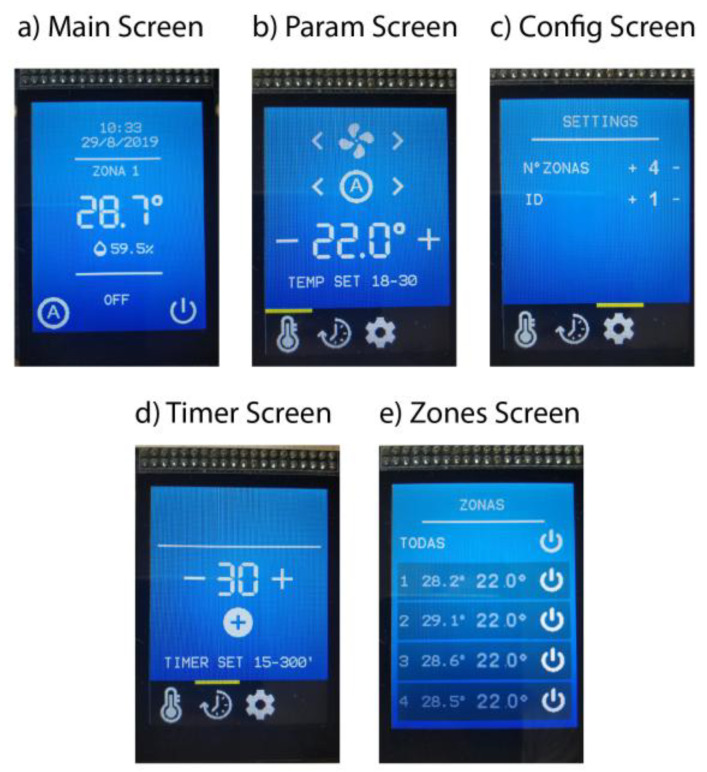
Screens designed for the user interaction: (**a**) Main screen, (**b**) Param screen, (**c**) Config screen, (**d**) Timer screen, and (**e**) Zones screen.

Five screens have been designed for the user interaction (Figure 5):

- Main screen: Indicates the system status providing date/time, room temperature. (Meas_Temp) and zone. It also includes the ON/OFF and setup buttons, etc.
- Param screen: Shows the configuration selected by the user for the room in terms of temperature (Set_Temp), fan velocity, etc.
- Config screen: Indicates the zone number.
- Timer screen: Configures several timers to program the air conditioning system.
- Zones screen: Shows the status of all zones or rooms, i.e., ON/OFF status, and the current (Meas_Temp) and set-point (Set_Temp) temperatures.

Each time the user touches the panel, the corresponding interrupt is generated and the XY position is obtained through the I2C bus. If the detected position is included within any defined region, the event associated to this button will be generated and attended. A timer (Timer_A0) has been activated and configured to trigger at 150 ms, to avoid the bounces that can be generated on the touch panel.

In order to reduce the energy consumption, the LCD touch screen will be turn off if it is not touched for 30 s. For that, the BLACKLIGHT pin has been connected to a PWM output of the microController. Thus, the panel can be turn off gradually giving a slowly shutdown effect. 

#### 2.3.2. Low-Power Deep Sleep Module (LPDS)

The LPDS (Low Power Deep Sleep) functionality has been implemented in the Nodes to minimize the energy consumption and power them from a 5 V battery. Concretely, in our case, a 5 V 2200 mA/h battery has been used for the designed prototype. When the Node is in deep sleep mode, the consumption obtained from the J12 measuring point of the CC3200-LAUNCHXL development board has been 0.1 mA.

The LPDS mode is activated when the LCD touch screen is turned off, i.e., after 30 s without touching the LCD panel.

The system will wake up from the LPDS mode every 30 s (through the PRCM interrupt), when the LCD touch screen is touched (through the GPIO interrupt) or when a MQTT message is received from the host bus.

In the active mode (LCD touch screen on), the TimerA0 is set to 1 s and during this period the pending tasks and events in the queues will be performed. Besides, the temperature and humidity acquisition, as well as the sending of synchronization MQTT messages will be also managed. In the LPDS mode (LCD touch screen off), the timer will be set to 30 s and the pending task will be performed. 

### 2.4. RPI Broker (Rasperry Pi)

The RPI Broker is based on the Raspberry Pi computer, which latest release includes on-board Wi-Fi, Bluetooth and USB capabilities, among other characteristics. Although older releases do not include the Wi-Fi connection, it can be provided by just using a common USB Wi-Fi adapter (e.g., Edimax EW-7811UN [39]).

In our case, the RPI Broker consists of a Raspberry Pi 2 model B+ [26]. Raspbian has been installed as operating system. Its main functions are:

1. To act as a wireless access point (WAP or AP), providing Wi-Fi coverage to the Controller and the Nodes in a secure way. For that, the Raspberry Pi 2 has been complemented with the Edimax EW-7811UN Wi-Fi card [39], which allows AP mode in WPA2 (Wi-Fi Protected Access 2). In addition, an access control based on the MAC (Media Access Control) address has been configured in order to control who is accessing to the Wi-Fi medium at all times.
2. To act as the MQTT Broker using the Eclipse Mosquito™ software [40]. The same SSL/TLS certificates used in the Nodes have been installed to enable encryption and access with username/password.
3. To provide the date/time using the NTP protocol. For this, an NTP client has been configured to obtain the correct date and time from an NTP server, and then an NTP server has been configured so that the RPI Broker itself provides the date and the time to the Nodes.

The OpenHAB package [32] has been installed and linked to the RPI Broker to provide an application programming interface (API) to the user through the mobile or web browser. In this way, the configuration and control of the air conditioning system might be done from anywhere.

### 2.5. Wi-Fi - MQTT Communication

This is one of the most important blocks in the designed system. As the CC3200 board incorporates the Wi-Fi interface inside, the sending and receiving of messages can be handle from the designed code using the libraries provided by Texas Instruments.

The MQTT protocol [28] is used for the communication. This protocol follows a star topology formed by a message broker and several clients. The broker manages the messages interchange in the network; meanwhile, the clients send periodically a request package and wait for the broker response. Each client is subscribed to a so-called “topic” or a set of them, from which messages are received or published to.

In our case, two topics have been defined, one for each direction of communication. In this way the communication between Controller and Nodes, and vice versa, is separated in two parts. If all modules were in a single topic, every time a message was published in the topic, everyone would receive that message and the microController would be interrupted to handle and process that message. This situation would become a bit critical in the Controller since it must be attending the serial bus.

All the Nodes are registered in the same topic. In this way, each of the Nodes knows the temperature and the configuration of each room and can connect/disconnect a certain area.

**Figure 6:**
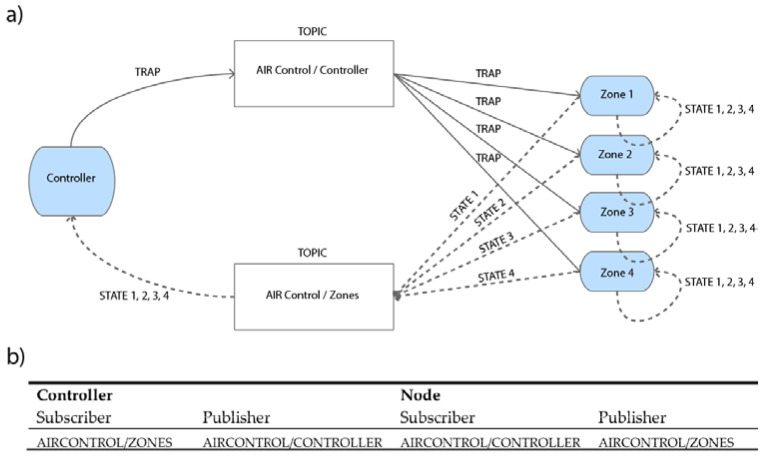
(**a**) Scheme of the topics assignment and (**b**) configuration.

Figure 6 shows the assignment and configuration of the topics of each module.

Two tasks have been created to accomplish the complete Wi-Fi–MQTT communication: Main and Wi-Fi–MQTT tasks. Once all the peripherals are initialized and the required variables (e.g., the SSID and the password) are read from the flash memory, the Main task starts the Wi-Fi connection by sending an event to the Wi-Fi–MQTT task. 

At this moment, everything required to connect to the wireless access point and provide an IP address is performed by the board. If there were any errors, the function would return an error code.

Once the connectivity has been established, the current date/time will be requested to the NTP server in order to be synchronized. 

Above the MQTT layer, it has been implemented by defining a message structure that will be inserted into the MQTT payload. The data structure should be as simple as possible and require the least possible resources of the microController. Therefore, it has been defined following a structure similar to the one used for the serial bus messages, avoiding data conversions and additional new data structure. Data are sent character by character, using the character “:” as delimiter between fields. 

Eight different types of messages have been defined, being identified through byte 2 of each frame (Table 1).

**Figure 7:**
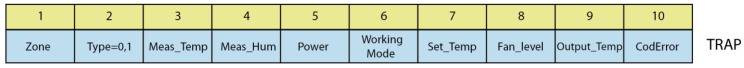
Structure of the trap message.

Trap messages (types 0 or 1) will be generated either if there is a change in the configuration of the air conditioning system or every 60 s, that is the time of the Keep-Alive timer implemented in the Controller. The structure of the trap messages is shown in Figure 7. It is composed of the following fields:

- Field 1 (Zone) indicates who is sending the message:
Controller→ 0Nodes→ 1, 2, 3 or 4.
- Field 2 (Type) indicates the communication situation:
Synchronized system→ 0System in synchronization process→ 1.
- Fields 3 and 4 (Meas_Temp and Meas_Hum) include the current temperature and humidity in each of the zones, including the Controller.
- Fields 5 to 10 show the configuration corresponding to the Node (or Controller) that sends the message. The Controller will always indicate the values obtained from the host bus corresponding to the main board.

The Setup and ACK Setup messages are used to configure the number of available zones. 

The Disconnect message has been defined to announce that an MQTT client has been disconnected from the system, which is called the Testament. When a Node (or Controller) is registered in the RPI Broker, a message is defined as Testament. In case of disconnection of one of them, this message will be sent to the topic so that the rest of Nodes and Controller will know. 

At the time they receive the Disconnect message, if it was from a Node it would be canceled from the system and it would not be possible to act on the corresponding zone. If the message came from the Controller, the Nodes would enter in the synchronism loss mode, and most of the functionalities and buttons would be canceled.

Finally, a request for the status of the Controller variables has been implemented in order to analyze and monitor the entire system. 

The Wi-Fi communication system has been also provided with the following security characteristics [28]:

- MQTT communication via SSL/TLS with certificates;
- RPI-Broker registration with username/password.

Before starting the communication with the RPI Broker, the date/time of the device must be configured, so that the certificate can be validated. If the date and time were not set, the MQTT session with SSL/TLS would never be established. For this reason, before opening the MQTT session, the date/time of the NTP server (i.e., Raspberry Pi) is retrieved. 

### 2.6. Temperature Control Algorithm

**Figure 8:**
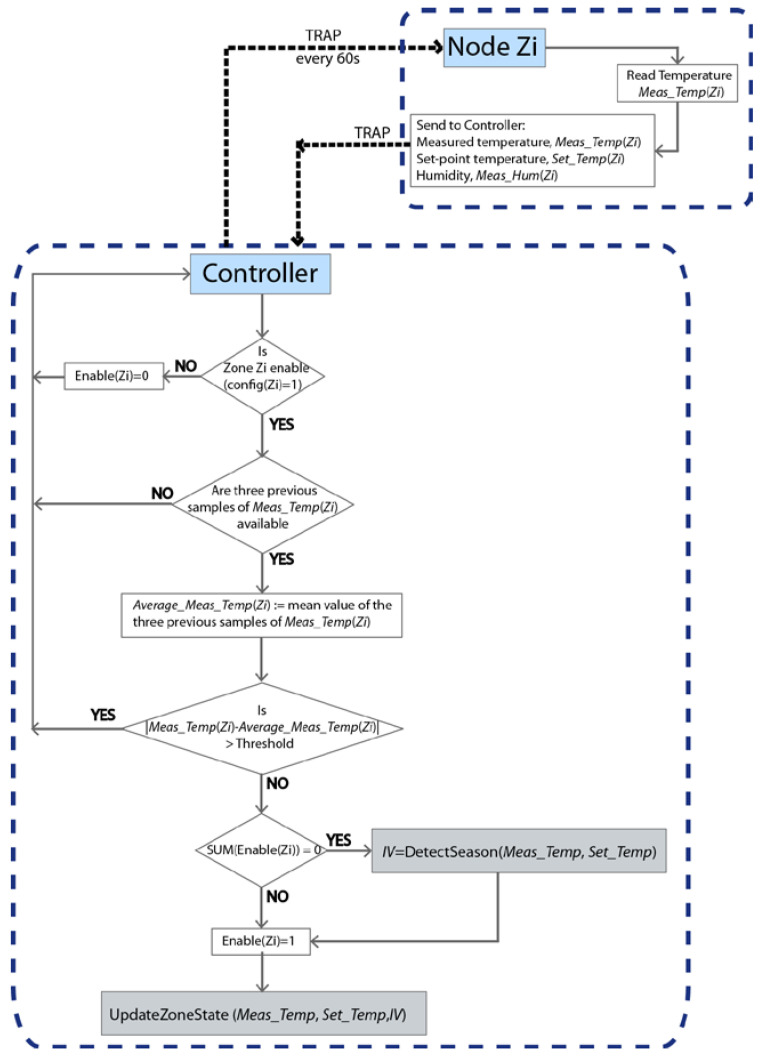
General flowchart of the temperature control algorithm.

The fan velocity (Fan_level) and the set-point temperature of the indoor machine (Output_Temp), as well as the opening of the vent registers in each of the zones, is managed by the temperature control algorithm. Figure 8 shows the general flowchart of the control process.

**Figure 9:**
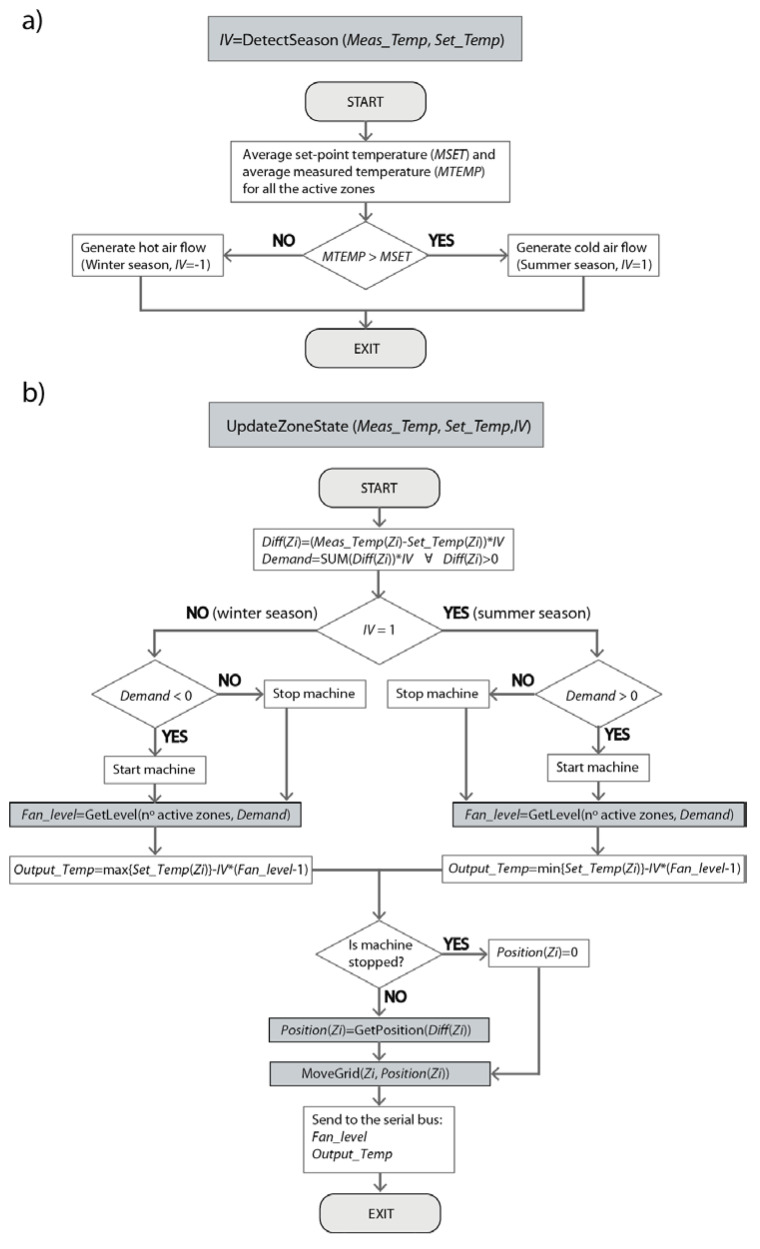
(**a**) Flowchart of the DetectSeason function; (**b**) flowchart of the UpdateZoneState function.

In the proposed scheme, a trap message is sent by the Controller to each of the Nodes every 60 s, and they return the current temperature (Meas_Temp), set-point temperature (Set_Temp) and the humidity in the respective zones. The variable config indicates if the zone is enabled (1) or not (0). If all the zones are turn off (config = 0), then there is nothing to do in the algorithm. In this status, the variable Enable is also set to 0 for all the zones. As soon as any zone is activated by the user (config = 1), and after checking the correct measurement of the temperature, the algorithm determines whether hot or cold air flow should be generated. The function DetectSeason is in charge of this task (see Figure 9a) and it is executed only once, at the initial moment that one of the zones is turned on by the user. If the average measured temperature for all the active zones (MTEMP) is higher than the average set-point temperature (MSET), then the air conditioning system should generate cold air flow (summer season). On the contrary, hot air flow should be generated (winter season). The variable IV has been defined to indicate to the system whether cold (summer, IV = 1) or heat (winter, IV = –1) should be generated.

In order to validate the correct temperature measurement, each new temperature sample is compared with the average value of the previous three temperature samples. In this way, if the difference value is higher than a defined threshold, the value will be taken as erroneous and not considered by the system. As a design condition, the threshold has been set as 3 °C, since it is very unlikely that a temperature change of that magnitude will occur in 60 s. 

Finally, the status of the machine (fan velocity and set-point temperature) and the position of the vent registers for each zone (see Figure 9b) are set by the UpdateZoneState function. This function is executed once the trap messages of each zone have been received or a time-out of 5 s has expired (e.g., due to the loss of a trap message or failure of a Node).

Initially, the differences between the measured and the set-point temperatures are obtained for each of the active zones and multiplied by the variable IV, Diff(Zi). Thus, a positive value of Diff(Zi) indicates that the desired temperature has not been reached in this zone yet. The sum of all the positive Diff(Zi) values multiplied by the variable IV is saved in the variable Demand. Negative Demand values correspond to the winter season, so the air conditioning system generates heat. In contrast, positive Demand values correspond to the summer season, in which the system generates cold. 

According to the specifications of Mitsubishi machines, three possible fan velocities (low, middle and high) are assigned, based on the absolute value of the Demand variable and the number of active zones (see Table 2). In the case of only one active zone, the fan velocity is set to the lowest level regardless of the value of the Demand variable. This avoids possible overpressures in the motor and the fan ducts. For more than one active zone, the thresholds for each fan level depend on the number of available zones, so any change in the zone configurations will imply the use of different threshold values. Concretely, the minimum and maximum thresholds for each possible configuration have been defined experimentally as 1 °C ×(Fan_level-1) × (Number of active zones) and 1 °Cx(Fan_level)x(Number of active zones), respectively. 

The set-point temperature of the indoor unit, Output_Temp, (see Section 2.2.1) is configured according to the relations shown in Table 3. Its value is based on the most restrictive set-point temperature of the active zones (i.e., the minimum set-point temperature in the summer season or the maximum set-point temperature in the winter season) and an additional factor that depends of the estimated fan velocity and the season (i.e., Fan_level and IV).

Finally, respecting the vent registers, four possible positions have been defined depending on the temperature differential in each zone (Table 4). The MoveGrid function is in charge of generating the PWM pulses and the appropriate timer to place the servo motor in the indicated position and the blades in the proper inclination.

If for some reason the Controller does not receive a trap message from one zone in 150 s (2.5 times the Hello timer), it will assume that there has been a problem in that zone and will disable it, recalculating the thresholds values. At the time the Controller receives two trap messages in a row again, it will enable the corresponding zone and recalculate the thresholds.

## 3. Results and Discussions

### 3.1. Technical Characteristics of the Multi-Zone System Prototype

As a result of the present investigation, a multi-zone system prototype has been implemented with the following technical characteristics:

- Controller:
  - CC3200 microController.
  - Power supply of 5 V obtained from the host bus through the designed line voltage adapter circuit (from 10 to 5 V).
  - Located on the ceiling, where the indoor unit is.
  - DHT22 temperature and humidity sensor [35] to measure the return air flow.
  - Panel of three status LEDs:
    ▪ Green: Flashing every second indicating normal operation;
    ▪ Yellow: Wi-Fi connection established;
    ▪ Red: Blinking for each frame detected on the host bus.
  - Maximum zone configurations:
    ▪ Basic: 4 PWM outputs for each of the vent registers;
    ▪ Possibility of adding 16 more outputs/zones by incorporating the PCA9685 16-channel module [37] to the CC3200;
  - Servo motor, MG996R [36], for the opening and closing of the vent registers.
  - Consumption:
    ▪ 75 mA normal operation;
    ▪ 2.5A pp with the 4 servo motors running.
  - Serial CLI interface at 115200 bauds for monitoring and provisioning tasks.
  - Wi-Fi/MQTT interface for communication with the RPI Broker and Nodes.
- Nodes:
  - Power supply of 5 V obtained from batteries.
  - Location of the Nodes:
    ▪ Node 1: Close to the original control panel, and powered by the host bus;
    ▪ Rest of Nodes: In each of the zones to be regulated and placing it well on the wall or on top of some furniture.
  - DHT22 temperature and humidity sensor [35].
  - Panel of three status LEDs, with the same functions that the ones in the Controller.
  - LCD touch screen, ER-type LCD TFTM028-4 [38].
  - Consumption:
    ▪ With the LCD touch screen:
      - 20 mA average;
      - 100 mA pp on start;
      - 0.2 mA (LPDS mode).
  - Serial CLI interface at 115200 bauds for monitoring and provisioning tasks.
  - Wi-Fi/MQTT interface for communication with the RPI Broker and Controller.
- Raspberry Pi:
  - Power supply through the micro-USB charger.
  - Model used: Raspberry Pi 2B with an Edimax EW-7811UN USB Wi-Fi adapter [39]. Latest releases (from model 3), incorporates the Wi-Fi interface.
  - Location: In a network outlet, preferably in the center of the house to provide good Wi-Fi coverage.
  - Installed services: SSH, NTP Server, NTP Client, Access Point server, Mosquito, Java, OpenHab.
  - Consumption:
    ▪ 320 mA normal operation;
    ▪ 450 mA pp on start.

The entire the developed system has been tested with the indoor FDUR-258A unit of Mitsubishi Electric Corporation and the associated main board (PJA505A092-A) and control panel (RDC-HE). It is important to highlight that, except for the CC3200 LaunchPad and the Raspberry Pi, all the components used in the design of the developed system are not completely exclusive, being able to use other temperature sensors, touch screen displays, and servo motors, etc.

**Figure 10:**
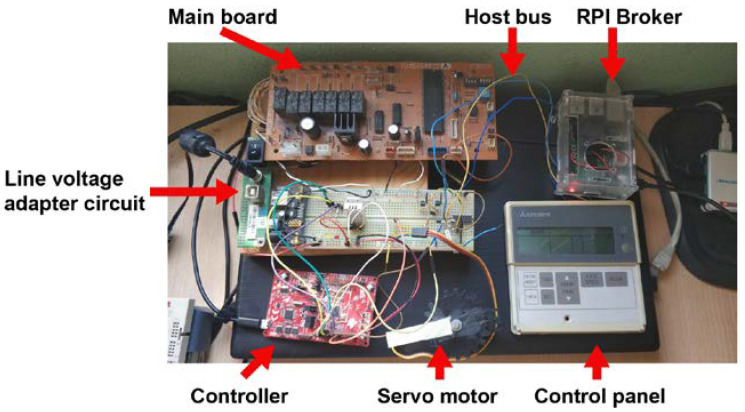
Assembly and main components associated to the Controller and the indoor unit.

**Figure 11:**
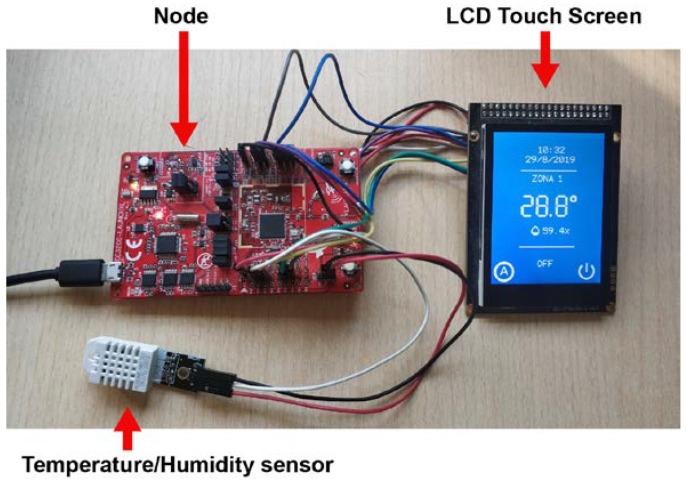
Assembly and main components associated to the Node.

Figure 10 shows the laboratory assembly corresponding to the indoor unit, which includes the main board, control panel, line voltage adapter circuit, and Controller with its DHT22 [35], status LEDs, and the servo motor [36]. Figure 11 shows the laboratory implementation of a complete Node, with all the different components.

The cost of all the electronic components used, assuming a house with three zones, is approximately €480, which is a much lower price than any commercial system.

### 3.2. Simulation of the Temperature Control Algorithm

Two different simulations have been carried out to verify the right performance of the temperature control algorithm in the summer and winter periods, respectively. The methodology adopted for both simulations is explained below.

**Figure 12:**
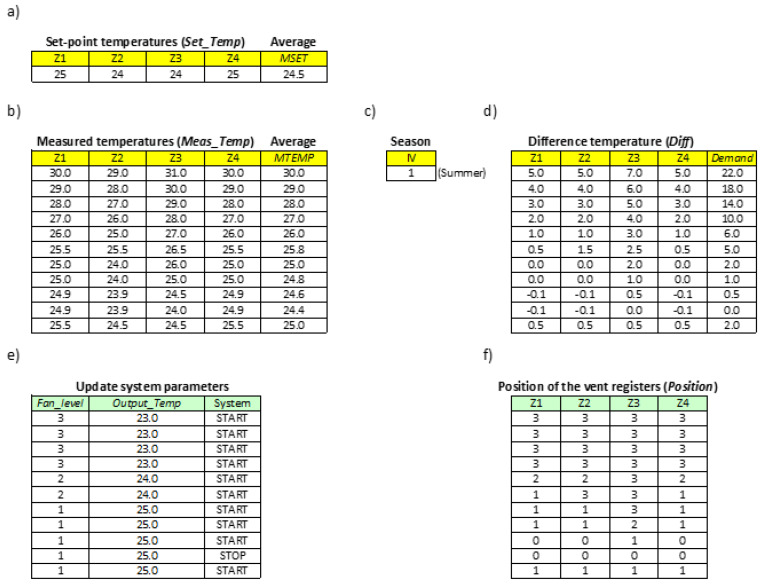
Example of simulation of the algorithm in the summer season. (**a**) Set-point temperatures, (**b**) measured temperatures, (**c**) season, (**d**) difference temperatures, (**e**) updating system parameters, (**f**) position of the vent registers.

**Figure 13:**
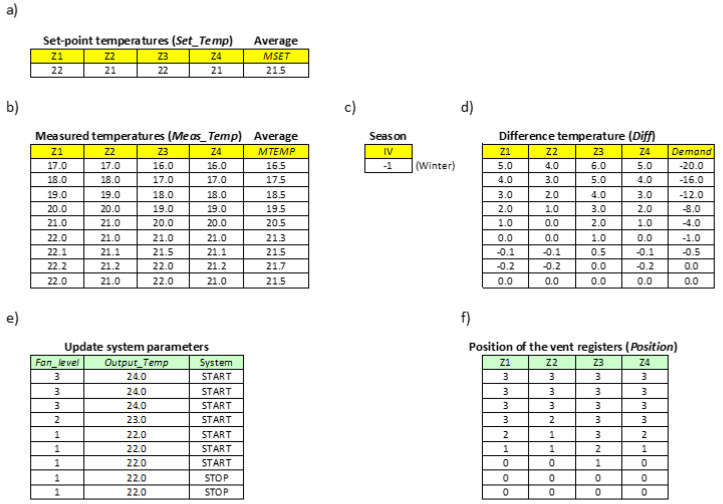
Example of simulation of the algorithm in the winter season. (**a**) Set-point temperatures, (**b**) measured temperatures, (**c**) season, (**d**) difference temperatures, (**e**) updating system parameters, (**f**) position of the vent registers.

For the simulation purposes, the four zones are enabled and configured each with different set-point temperatures (Set_Temp) (Figure 12a and Figure 13a). The initial temperatures measured in each of the zones, i.e., measured temperatures (Meas_Temp) are also assigned (Figure 12b and Figure 13b) and the average value is calculated (MTEMP). 

Comparison of the initial value of MTEMP with the average value of the set-point temperatures (MSET) determines whether to generate cold (summer season, IV = 1) or hot air (winter season, IV = −1) (Figure 12c and Figure 13c).

From this moment on, for each of the temperatures measured in each of the zones (Figure 12b and Figure 13b), the temperature control algorithm (Figure 9b) will obtain the variables Diff(Zi) and Demand (Figure 12d and Figure 13d), and will update the status of the indoor unit (Figure 12e and Figure 13e) and the vent registers (Figure 12f and Figure 13f). Concretely, the set-point temperature (Output_Temp) and the fan velocity (Fan_level) of the indoor unit (see Table 2 and Table 3), as well as the position of the vent registers (see Table 4), will be configured.

To simulate the complete process and observe how the different algorithm variables change, different measured temperatures have been assigned progressively in increasing or decreasing order (Figure 12b and Figure 13b), depending on whether hot or cold air is generated, respectively.

Figure 12 shows an example of a simulation of the algorithm in the summer period. The temperatures in each of the zones have been varied simulating a period in summer and it can be seen how the cold air demand decreases as temperatures reach the configured ones. The status of the machine is indicated through the System variable as START/STOP. When there is no demand for cold air, the machine stops. When the system requires again cold air flow, the machine restarts automatically.

Figure 13 shows the behavior of the algorithm in the winter season, where hot air must be generated. In this period, the hot air flow demand is established with negative values (variable IV). The functioning of the algorithm is similar to the one explained for the summer period

**Figure 14:**
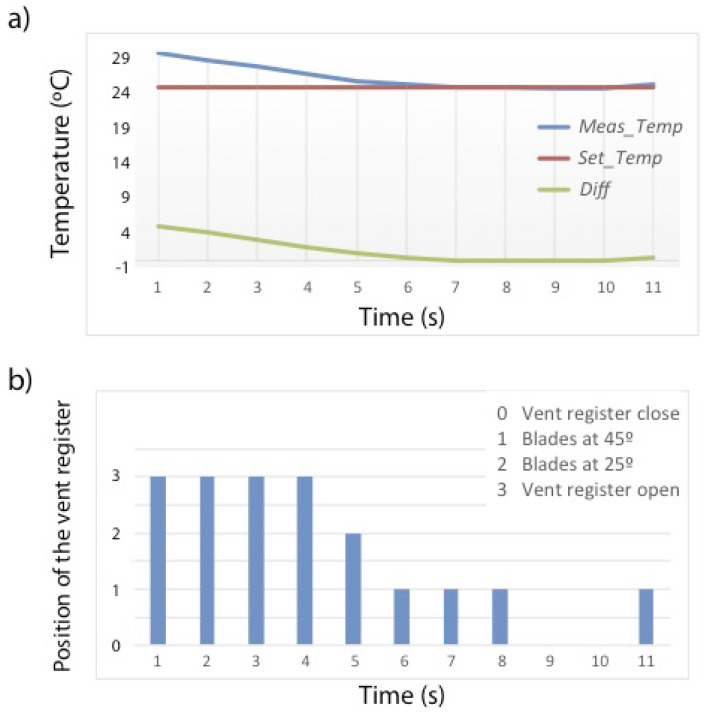
Example of the algorithm performance in the summer season for Zone 1. (**a**) Variation of the current temperature (blue line) together with the configured one (red line) and the air flow demand (green line). (**b**) Opening level of the blades according to the flow demand.

In Figure 14a, the variation of the temperature in Zone 1 is shown (blue line) together with the configured one (red line). The air flow demand (green line) is also adjusted depending on the temperature difference. At the beginning, a large amount of cold air demand is required, so the total opening of the vent register is ordered, placing it in position 3. As the demand decreases, the blades are closing more and more. Figure 14b shows the opening level of the blades and how they are adjusted according to the air flow demand. At the moment the demand is zero, that is, when the current temperature (Meas_Temp) is below the desired temperature (Set_Temp), then the vent register will be closed completely. In the case that no vent register is open at this time, the machine will automatically shut down since there is no demand, avoiding the problem of the pressure generated by the fan in the air ducts, which might eventually damage the system.

### 3.3. Experiments in Energy Saving

**Figure 15:**
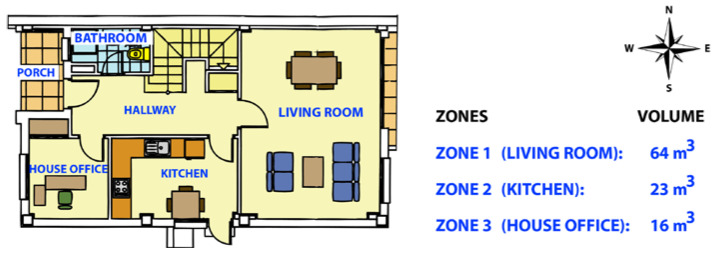
Map of the control zones and their respective volumes.

Finally, two different real experiments were carried out in order to study and compare the energy consumption with single and multi-zone HVAC systems. The experiments were performed in a two-floor house located in Alicante (southeast Spain) over eight days, between 2 April and 5 May 2020. Concretely, the ground floor, prepared with the installation of single-zone air conditioning, was divided in three control zones: Zone 1, living room; Zone 2, kitchen; and Zone 3, house office. Figure 15 shows the distribution of the ground floor along with the volume of the selected zones. The three zones are facing south. The walls are of brick construction with expanded polystyrene insulation, and the roof is covered with ceramic tiles and is uninsulated. 

In order to carry out these experiments, the Controller has been programmed to operate in normal or listen-only mode, depending on whether it is enabled or not, respectively. In this way, when the Controller is disabled, it only monitors the signals of the host bus and each of the Nodes, but does not act on the system.

Both real experiments followed a common methodology to estimate the energy consumption in the single-zone and multi-zone modes. 

In the first step, the single-zone system was tested disabling the Controller. With this configuration, the system worked without individual temperature control for each of the zones and without any control on the vent registers. Subsequently, the Controller was enabled and then, the multi-zone system was activated. In this configuration, the control of the entire system was taken by the Controller, receiving from each Node the temperature of each zone and acting on the indoor unit (Output_Temp and Fan_level) and the vent registers of each room.

To measure energy consumption, an energy meter based on the CC3200 microController has been designed and implemented. The voltage and current are sampled at 1 kHz and the energy consumption data is sent every 60 s to the RPI Broker, where the information is saved in a log file for further analysis. The developed energy meter has been calibrated with a Fronius Smart Meter 63A-1 (energy accuracy of 1%) ([41]), obtaining a mean relative error of 1%.

In the first test, the energy consumption required by the system to reach the desired temperature (Set_Temp) in the whole house (i.e., in all the zones) was analyzed. The returned air, outside and set-point temperatures (Return_Temp, Ext_Temp and Set_Temp) were monitored, as well as the temperature measured in each zone (Meas_Temp). The status of the machine (START/STOP), the fan velocity and the energy consumption are also represented over time. This is similar to the test carried out by Li et al. [25]. 

**Figure 16:**
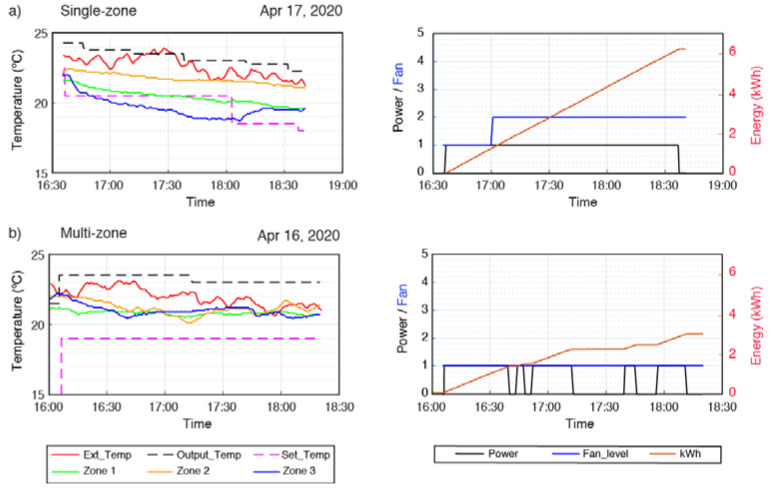
Variation of temperatures and energy consumption. (**a**) Single-zone and (**b**) the proposed multi-zone system.

First, cold air generation was investigated, setting the desired temperature for all the zones at 21 °C. In the single-zone mode (Figure 16a), two hours were required to reach the desired temperature in all the zones. The energy consumption of the system over these two hours was of 6.2 kWh. The first zone to reach the desired temperature was Zone 3 (house office), because it is the smallest one and because it is right in front of the interior machine. The second one was Zone 1 (living room), since it is the one with the largest vent register. Zone 2 (kitchen) reached the desired temperature almost at the end of the studied period, what produced that the system was power on over the two hours. Although Zone 2 is not the zone with the highest volume, it is the one with the smallest vent register and it is surely the zone with the highest thermal losses. In the single-zone mode, the temperature of each zone is not considered by the system and the vent registers remains always open. Thus, while almost two hours were needed to reach the desired temperature in Zone 2, in other zones such as Zone 3 the temperature was below 19 °C during some intervals. Therefore, it is clear that the absence of a temperature control produces an uncomfortable situation in all the zones.

In the case of the multi-zone mode (Figure 16b), the system was evaluated during a period of time equal to the one used in the single-zone mode, just for comparison purposes. The energy consumption over the two hours was of 3.5 kWh, which implies a reduction of 44% compared to the single-zone system. All three zones reached the desired temperature in approximately 45 min. But the most important thing in regard to user comfort is that once the desired temperature was reached, it remained stable throughout the rest of the time.

Regarding the hot air generation, the desired temperature was set to 22 °C and the energy consumption was of 3.8 and 3 kWh for the single and multi-zone modes, respectively. This implies an energy saving of 22%. 

In the second test, the energy consumed by the air conditioning system to reach the temperature of 22.5 °C in each of the zones was studied. First, the system was used in single-zone mode and then, applying the designed multi-zone system, each of the rooms was cooled sequentially: Zone 1, Zone 2 and Zone 3. This is similar to the test carried out by Watts et al. [24] to heat a four-zone house.

In the single-zone mode, the initial temperatures for each zone were 24.3, 25.3 and 24.4 °C for Zone 1, Zone 2 and Zone 3, respectively. As was noted previously, in the first test the absence of temperature control produces the user discomfort. In fact, after four hours and 40 min, the final temperatures for each zone were 21.3, 23.5 and 20.8 °C for Zone 1, Zone 2 and Zone 3, respectively. While in Zone 2 it was still hot, in Zones 1 and 3 it was cold, in reference to the desired temperature. The energy consumption was of 16.5 kWh over the analyzed time, even when the desired temperature was not reached in Zone 2.

In the case of the multi-zone mode, the initial temperatures were very similar to those of the house in the single-zone study. That is 24.3, 25.4 and 24.7 °C for Zone 1, Zone 2 and Zone 3, respectively. The different zones reached the desired temperature (i.e., 22.5 °C) in 45, 49 and 13 min, respectively. Zone 2 (kitchen) takes the longest time to reach the desired temperature, perhaps due to the small size of the vent register and the thermal losses commented in the previous experiment. Regarding the energy consumption required to individually cold each of the zones; it was of 2.6, 2.6, and 0.6 kWh, for Zones 1, 2, and 3, respectively.

**Figure 17:**
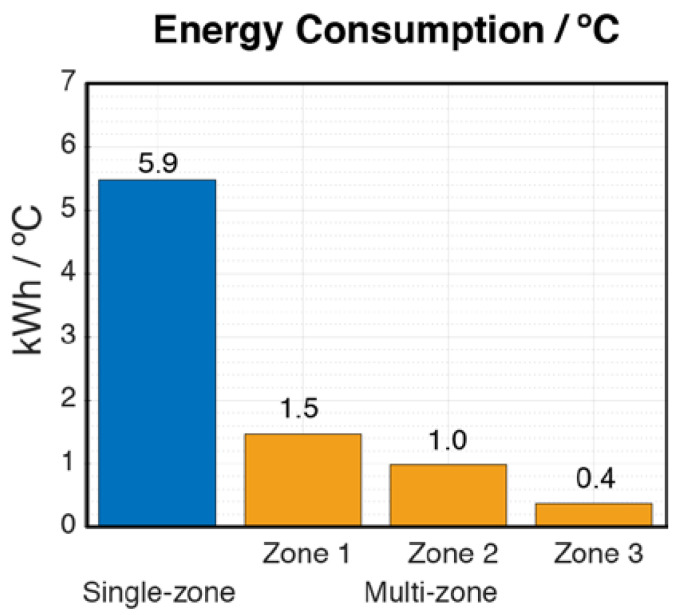
Energy consumption/°C for the whole house (in single-zone mode) and for each of the zones (in multi-zone mode).

Figure 17 compares the energy consumption per degrees Celsius obtained with the single-zone system for the entire house with that obtained by the multi-zone system for each of the rooms. In the single-zone mode, the mean of the differences between the final and initial temperatures of each room was used for the calculation.

The energy saving for each of the zones was 75%, 83%, and 94% for Zones 1, 2, and 3, respectively, compared to the single-zone system.

In the work of Rajith et al. [13], an automatic control system was proposed to act on several individually controlled indoor air conditioning units. Their experiments were performed in an office space during summer season, achieving 20%–40% saving in energy consumption approximately. A room-level zoning system was presented by Sookor et al. [10] for heating and cooling homes by conditioning only occupied spaces. In this case, an energy reduction of 20.5% was obtained for an eight-room, single story, 1200 square foot residential building analyzed over 20 days. Finally, in the work of Watts et al. [24], a multi-zone HVAC control was applied to heat a two-story house over three days. In this case, energy savings between 50% and 94% was obtained comparing the energy consumption for the whole house with the one for each of the selected zones. 

Therefore, the effectiveness of the developed system exceeds that of other similar systems implemented in residential buildings to space cooling. Compared with the single-zone system, an energy saving between 75 and 94% is obtained when only one of the zones is selected, and 44% when the whole house is cooled. Besides, as it has been demonstrated in the accomplished tests, the user comfort is considerably improved by the developed multi-zone system, in comparison to the single-zone one. 

## 4. Conclusions

In this work, a low-cost IoT solution has been adopted to transform an existing single-zone HVAC installation into a multi-zone HVAC system. The developed WSN is based on the Raspberry Pi computer and the low-cost and low-power CC3200 microController, working with Wi-Fi connectivity and the MQTT protocol.

As a novelty compared to other previous systems found in the literature for residential buildings, the developed system controls the vent registers (with four different opening states), and the fan velocity and the set-point temperature of the indoor unit. For that, the prototype is connected directly to the communication bus of the existing HVAC system.

Simulations and real experiments have been carried out to analyze the performance of the complete system and evaluate the energy consumption compared with the single-zone mode. In this regard, an energy saving between 75% and 94% is provided by the designed system when only one of the zones is selected, and 44% when the whole house has to be cooled. Thus, the proposed system improves the results obtained with other similar systems implemented in residential buildings to space cooling. 

Apart from saving energy, it is important to highlight how the user comfort is improved by the multi-zone system, reaching the desired temperature earlier in each zone and keeping it stable over time.

For the equipment implementation, commercial off-the-shelf components and open-source software have been used, so any researcher can reproduce the proposed system and adapt it to their own requirements (e.g., a different number of zones, a different temperature control algorithm, etc.). The installation is accessible for any type of house. Additional masonry or electricity works are not required.

A first prototype has been implemented to test the performance of the developed WSN architecture. For future work, additional improvements could be carried out in the implementation process in order to reduce the consumption of the designed equipment. Designing our own circuit based on the CC3200 microController instead of using the CC3200 LaunchPad development board might reduce the power consumption. It might also be reduced using other low-power components (e.g., temperature sensors [42], touch screen displays, etc.). Besides, alternative temperature control algorithms based on artificial neural networks, and fuzzy logic, etc., could be also tested. 

## Figures and Tables

**Table 1:** Type of messages.

Type of Messages	Byte 2
Synchronized system	0
System in synchronization process	1
Setup zones	5
ACK (Acknowledgement) Setup zones	6
Disconnect	16
Request variables (VARS)	253
Respond variables (VARS)	254
Send “Hello”	255

**Table 2:** Defined fan levels for the air flow demand control.

Fan_Level		4 Zones			3 Zones			2 Zones			1 Zone
1 (low)	+0<=	|Demand|	<=4	0<=	|Demand|	<=3	0<=	|Demand|	<=2	0<=	|Demand|
2 (middle)	+4<	|Demand|	<=8	3<	|Demand|	<=6	2<	|Demand|	<=4		
3 (high)	+8<	|Demand|		6<	|Demand|		4<	|Demand|			

**Table 3:** Configuration of the set-point temperature of the indoor unit (Output_Temp).

Relation	Season
min{Set_Temp(Zi)}-IVx(Fan_level-1)	Summer
max{Set_Temp(Zi)}-IVx(Fan_level-1)	Winter

**Table 4:** Positions of the vent registers.

Position	Description	Temperature Differential		
0	Vent register close		Diff(Zi)	<0
1	Blades at 45°	0<=	Diff(Zi)	=<0.5
2	Blades at 25°	0.5<	Diff(Zi)	=<1
3	Vent register open	1<	Diff(Zi)	

## Abbreviations

It describe the abbreviations and variables used throughout this paper.

**Abbreviations**	**Description**
HVAC	Heating, Ventilation and Air Conditioning
CAV	Constant Air Volume
VAV	Variable Air Volume
WSN	Wireless Sensor Network
IoT	Internet of Things
MQTT	Message Queuing Telemetry Transport
RPI Broker	Raspberry Pi computer
NTP	Network Time Protocol
OpenHab	Open Home Automation Bus
SSL	Secure Sockets Layer
PHP	Personal Home Page
SSID	Service Set Identifier
PWM	Pulse-Width Modulation
LCD	Liquid Crystal Display
I^2^C	Inter-Integrated Circuit
ACK	Acknowledgement
CSS	Code Composer Studio
SDK	Software Development Kit
RTOS	Real-Time Operating System
CLI	Command Line Interface
PRCM	Power Reset and Clock Module
LPDS	Low-Power Deep Sleep
WAP or AP	Wireless Access Point
WPA”	Wi-Fi Protected Access 2
API	Application Programming Interface
TLS	Transport Layer Security
WLAN	Wireless Local Area Network
UART	Universal Asynchronous Receiver-Transmitter
TFT	Thin Film Transistor
SPI	Serial Peripheral Interface
MAC	Media Access Control
	
**Variables**	**Description**
Output_Temp	Selected temperature at the air outlet of the indoor unit
Fan_level	Selected ventilation velocity in the indoor air conditioning unit
Return_Temp	Temperature measured in the returned air of the ceiling
Set_Temp	Set-point temperature in each of the zones
Meas_Temp	Temperature measured in each of the zones
Meas_Hum	Humidity measured in each of the zones
MTEMP	Average measured temperature for all the active zones
MSET	Average set-point temperature
IV	Generation of cold (summer, IV = 1) or hot (winter, IV = −1) air flow
